# Circulating tumour cell detection: a direct comparison between the CellSearch System, the AdnaTest and CK-19/mammaglobin RT–PCR in patients with metastatic breast cancer

**DOI:** 10.1038/sj.bjc.6605472

**Published:** 2009-12-01

**Authors:** I Van der Auwera, D Peeters, I H Benoy, H J Elst, S J Van Laere, A Prové, H Maes, P Huget, P van Dam, P B Vermeulen, L Y Dirix

**Affiliations:** 1 Translational Cancer Research Group (Laboratory of Pathology, University of Antwerp/University Hospital Antwerp, Oncology Centre, General Hospital Sint-Augustinus), Antwerp, Belgium; 2 Laboratory for Molecular Biology, Labo Lokeren, Campus RIATOL, Antwerp, Belgium

**Keywords:** circulating tumour cells, breast cancer, CellSearch System, RT–PCR, AdnaTest

## Abstract

**Background:**

The detection, enumeration and isolation of circulating tumour cells (CTCs) have considerable potential to influence the clinical management of patients with breast cancer. There is, however, substantial variability in the rates of positive samples using existing detection techniques. The lack of standardisation of technology hampers the implementation of CTC measurement in clinical routine practice.

**Methods:**

This study was designed to directly compare three techniques for detecting CTCs in blood samples taken from 76 patients with metastatic breast cancer (MBC) and from 20 healthy controls: the CellSearch CTC System, the AdnaTest Breast Cancer Select/Detect and a previously developed real-time qRT-PCR assay for the detection of *CK-19* and *mammaglobin* transcripts.

**Results:**

As a result, 36% of patients with MBC were positive by the CellSearch System, 22% by the AdnaTest, 26% using RT–PCR for *CK-19* and 54% using RT–PCR for *mammaglobin*. Samples were significantly more likely to be positive for at least one mRNA marker using RT–PCR than using the CellSearch System (*P*=0.001) or the AdnaTest (*P*<0.001).

**Conclusion:**

We observed a substantial variation in the detection rates of CTCs in blood from breast cancer patients using three different techniques. A higher rate of positive samples was observed using a combined qRT-PCR approach for *CK-19* and *mammaglobin*, which suggests that this is currently the most sensitive technique for detecting CTCs.

Metastasis is the leading cause of breast cancer-related deaths, but early spread of tumour cells usually remains undetected even by high-resolution imaging technologies. Traditional prognostic factors do not accurately predict which patients will eventually relapse after primary treatment, and provide only limited information on the effectiveness of adjuvant treatment. Accumulating reports show that the detection of circulating tumour cells (CTCs) in body fluids has considerable potential to improve the clinical management of patients with breast cancer (Cristofanilli *et al*, 2004, 2005; Budd *et al*, 2006; Hayes *et al*, 2006; Wong *et al*, 2006; Benoy *et al*, 2006a; Rack *et al*, 2009). Over the past few years, different approaches for the detection, enumeration and isolation of CTCs in blood have been developed. Immunocytochemical analysis is usually used in combination with density-gradient centrifugation (Balic *et al*, 2005; Muller *et al*, 2005; Wiedswang *et al*, 2006), size filtration (Kahn *et al*, 2004; Wong *et al*, 2006) or flow cytometry (Meng *et al*, 2004; Allan *et al*, 2005) to enrich tumour cells before their detection. In addition, nucleic-acid-based approaches for cell detection have been described (Lambrechts *et al*, 1999; Smith *et al*, 2000; Aerts *et al*, 2001; Stathopoulou *et al*, 2002; Benoy *et al*, 2004). There is, however, substantial variability with regard to the rates of positive samples using existing techniques. Lack of standardisation of technology hampers the implementation of CTC measurement in clinical routine practice.

This study was designed to directly compare three techniques for detecting CTCs in the blood of patients with metastatic breast cancer (MBC). The first technique is called the CellSearch System (Veridex LLC, Raritan, NJ, USA), developed to automatically enrich and immunocytochemically detect CTCs from peripheral blood (Allard *et al*, 2004), and is currently the only instrument with regulatory approval for routine clinical use in MBC patients. This system consists of CellSave Preservative tubes, preventing CTC degradation for up to 96 h; the CellSearch CTC kit, a pre-packaged kit for the isolation and identification of CTCs; CellSearch control cells for assuring proper performance on a daily or run-to-run basis; the CellTracks AutoPrep system for automatically adding reagents and the CellTracks Analyzer II, a semi-automated microscope for scanning and reading results. Epithelial cells are immunomagnetically separated and fluorescently labelled, and nucleated (DAPI+) cells with the EpCAM+, cytokeratin (CK) 8/18/19+ and CD45− phenotype are counted as CTCs. Riethdorf *et al* (2007) validated the CellSearch System in a multi-centre study and concluded that the system allows the reliable detection of CTCs in blood and is suitable for the routine assessment of MBC patients in a clinical laboratory. In a study by Cristofanilli *et al* (2004, 2005), 177 patients with MBC were tested for the presence of CTCs using the CellSearch System. The study concluded that detection of CTCs before initiation of first-line therapy in patients with MBC is highly predictive of progression-free and overall survival. Furthermore, it was recently shown that CTCs persisting after cytostatic, endocrine and zoledronate treatment can be observed in a relevant number of clinically recurrence-free breast cancer patients. A longer follow-up of these patients will provide further insight in their prognostic relevance and show whether they can be used for real-time tumour phenotyping or serve as treatment target (Rack *et al*, 2009). The second CTC detection method is the AdnaTest Breast Cancer Select/Detect (AdnaGen AG, Langenhagen, Germany), in which immunomagnetic separation is followed by a multiplex RT–PCR for the tumour-associated transcripts *HER2*, *Muc-1* and *GA773-2*. This method has been shown to be a highly sensitive approach with a detection limit of two tumour cells (Zieglschmid *et al*, 2005). Using this technique, CTCs were detected in 69% of patients with MBC (Zieglschmid *et al*, 2007a). The clinical validation of this diagnostic system in breast cancer has not yet been reported. However, in colorectal cancer, the presence of CTCs detected by the AdnaTest Colon Cancer Select/Detect technique in the peripheral blood collected before surgery, as well as in follow-up samples, provided prognostic information (Zieglschmid *et al*, 2007b).

The final method of analysis is a multimarker real-time qRT-PCR assay. This assay has been previously developed in our laboratory for the detection of *CK-19* and *mammaglobin* transcripts in peripheral blood and bone marrow samples of patients with breast cancer (Benoy *et al*, 2004). CK-19 is expressed in the majority of breast carcinomas (Bartek *et al*, 1985) and has been extensively used as a marker for CTC (Slade *et al*, 1999; Smith *et al*, 2000; Aerts *et al*, 2001; Stathopoulou *et al*, 2003; Benoy *et al*, 2004; Ring *et al*, 2005). The specificity of *mammaglobin* for the detection of breast cancer cells in haematopoietic products has been evaluated in several studies (Leygue *et al*, 1999; Zach *et al*, 1999; Suchy *et al*, 2000; Corradini *et al*, 2001; Silva *et al*, 2002; Lin *et al*, 2003). In a recent study, we observed that the detection of *CK-19* and *mammaglobin* transcripts in bone marrow samples from untreated patients with breast cancer was superior to immunocytochemistry in predicting patients’ prognosis (Benoy *et al*, 2006a, 2006b). Kaplan–Meier survival analysis showed a markedly reduced overall survival among patients with elevated *CK-19* and *mammaglobin* mRNA levels in bone marrow. However, the presence of CTC in blood had no impact on patients’ overall survival.

## Materials and methods

### Patients and sample collection

We obtained peripheral blood samples from 76 patients with MBC and from 20 healthy volunteers. All patients gave informed consent for the use of their blood specimen, and examination of blood samples was carried out after approval from the institutional review board of the General Hospital Sint-Augustinus (Antwerp, Belgium). Blood samples were taken from 60 patients receiving treatment for MBC (treated patients) and from 16 patients who presented themselves at our clinic with untreated MBC (untreated patients). Treated patients received different cytostatic treatments mostly containing taxanes, vinorelbine, anthracyclines or capecitabine (*N*=40), endocrine therapy (*N*=17) or trastuzumab alone or in combination with other treatments (*N*=18). Most patients were extensively pretreated. Samples were taken at least 3 weeks after previous chemotherapy administration, and all patients were sampled only once. The median age of the control population was 39 (range, 25–54) years and 62 (range, 34–85) years in the breast cancer population. Clinicopathological variables were entered in a database and are listed in Table 1. Disease status was assessed using the Response Evaluation Criteria in Solid Tumours Group (RECIST) criteria without knowledge of the patients’ CTC results (Therasse *et al*, 2000). Disease status was subsequently dichotomised into either progressive (RECIST: PD) or non-progressive disease (RECIST: SD, PR, CR). The study was conducted in a double-blinded manner: the patients’ disease status was not known to individuals who performed the blood assays (IVdA, DP, IB, HE and SVL) and assay results were not known to the individual who recorded the disease status (PVD, PH, AP and LD). The AdnaTest Breast Cancer Select/Detect technique was performed independently of the other two CTC assays by the Laboratory for Molecular Biology (IB) (Labo Lokeren, Campus RIATOL, Antwerp, Belgium), which was blinded to the other assay results.

### CellSearch CTC test

Peripheral blood (10 ml) was collected from each donor into CellSave blood collection tubes (Immunicon Inc., Huntingdon Valley, PA, USA), which are evacuated blood draw tubes containing EDTA and a cellular preservative, and processed within a maximum of 72 h after blood drawing (at room temperature). Circulating tumour cells were enumerated with the CellSearch System (Veridex, Raritan, NJ, USA) as described by Allard *et al* (2004). Briefly, 7.5 ml of blood was gently mixed with 6.5 ml of dilution buffer, centrifuged (800 **g**, 10 min, gentle deceleration) at room temperature and transferred into the CellTracks AutoPrep system. After aspiration of the plasma and dilution buffer layer, anti-EpCAM-antibody-coated ferrofluids were added. After incubation and magnetic separation, unbound cells and remaining plasma were removed, and ferrofluid-labelled cells were re-suspended in buffer, permeabilised and fluorescently labelled using phycoerythrin-conjugated anti-cytokeratin antibodies recognising cytokeratins (predominantly cytokeratins 8, 18 and 19) to specifically identify epithelial cells; with an antibody against CD45 conjugated with allophycocyanin to identify WBC and a with nuclear dye (4′,6-diamidino-2-phenylindole, DAPI) to fluorescently label cell nuclei. The sample was transferred automatically to a cartridge in a MagNest, in which immunomagnetically labelled cells move to the surface caused by the strong magnetic field of the MagNest device. The MagNest was placed on the CellTracks Analyzer II, a four-colour semi-automated fluorescence microscope, and image frames covering the entire surface of the cartridge for each of the four fluorescent filter cubes were captured. The captured images containing objects that met predetermined criteria were automatically presented in a web-enabled browser from which final selection of cells was carried out by the operator. The main criteria for an object to be defined as a CTC included a round-to-oval morphology, a visible nucleus (DAPI+), positive staining for cytokeratin and negative staining for CD45. Results of cell enumeration were expressed as the number of cells per 7.5 ml of blood, and a cutoff of ⩾2 CTC was chosen to define the test as positive. Each sample was analysed independently by two readers (HE and PV). Questionable interpretations were evaluated again until consensus was reached.

### AdnaTest Breast Cancer Select/Detect

Blood (2 × 5 ml) samples were taken using AdnaCollect blood collection tubes (AdnaGen, Langenhagen, Germany) and immediately placed on ice. For each donor, the AdnaTest Breast Cancer Select/Detect technique was used on two separate blood samples, according to the manufacturer's instructions.

#### AdnaTest Breast Cancer Select

BreastSelect Beads (100 *μ*l) were added to 5 ml of blood and incubated for 15 min at room temperature (5 r.p.m.). After incubation, cells were repeatedly washed with PBS and lysed by adding a Lysis/Binding buffer (AdnaGen). The supernatant was recovered.

#### AdnaTest Breast Cancer Detect

mRNA was subsequently recovered by a magnetic separation using Oligo(dT)_25_ Dynabeads. The total mRNA/bead mixture (29.5 *μ*l) was reverse transcribed using 0.5 *μ*l of RNase inhibitor (40 U *μ*l^−1^; Promega, Madison, WI, USA), 4 *μ*l of RT buffer, 4 *μ*l of dNTPs and 2 *μ*l of Sensiscript Reverse Transcriptase (Qiagen, Valencia, CA, USA). Reverse transcription was performed in a one-step reaction (60 min at 37°C, 5 min at 93°C). The mixture was then chilled down on ice and stored at −20°C.

For the analysis of tumour-associated mRNAs, a multiplex PCR was carried out. The primer mixture consisted of four specific primer pairs for the amplification of three tumour markers (*Muc-1*, *HER2* and *GA733-2*) and one housekeeping gene (*Actin*). PCR analyses were carried out in a final volume of 50 *μ*l PCR mixture, containing 8 *μ*l of cDNA, 4 *μ*l primer mixture (PrimerMix BreastDetect; AdnaGen), 25 *μ*l of Hot Star Taq Master Mix (Qiagen) and 13 *μ*l of distilled water. PCR analyses were performed as follows: pre-denaturation at 95°C for 15 min, followed by 35 cycles of denaturation at 94°C, annealing at 60°C for 1 min, extension at 72°C for 1 min and a final extension step at 72°C for 10 min.

For negative controls, mRNA and cDNA were replaced by water in the reverse transcription and PCR experiments.

#### Evaluation

Visualisation of data was carried out using the BioAnalyzer 2100 (Agilent Technologies, Santa Clara, CA, USA) on a DNA 1000 LabChip. For interpretation of the test result, a fragment of the control gene actin had to occur in each sample (internal PCR control). The AdnaTest was considered to be positive if a PCR fragment of at least one tumour-associated transcript was clearly detected (peak concentration of >0.30 ng *μ*l^−1^). Peaks that were not detected at the above setting were negative (concentration <0.15 ng *μ*l^−1^). Peaks with an intermediate concentration of 0.15–0.30 ng *μ*l^−1^ were considered to be inconclusive. For a participant in this study to be diagnosed as positive for CTC in blood, both blood samples had to have a positive result by the AdnaTest.

### Quantitative multimarker RT–PCR assay

#### Total RNA extraction

Blood (9 ml) was collected from each donor into VenoSafe EDTA blood collection tubes (Terumo Europe, Leuven, Belgium). First, blood was passed through a LeukoLOCK filter (Ambion/Applied Biosystems, Foster City, CA, USA), which captures the total leukocyte population. RNA in cells captured on the filter was then stabilised with Ambion RNAlater Solution (Ambion/Applied Biosystems) and stabilised cells were stored on the filter at −20°C until further use. Total RNA was purified using the bead capture technology of the LeukoLOCK system (Ambion/Applied Biosystems) and quantified using the NanoDrop ND-1000 (Thermo Fischer Scientific, Wilmington, DE, USA).

#### RT–PCR

RNA (2 *μ*g) was reverse transcribed in a final volume of 100 *μ*l using a High Capacity cDNA Archive kit (Applied Biosystems). All PCR reactions were performed on a 7900HT Fast Real-time PCR System (Applied Biosystems). Fluorogenic probes and primer sets for *CK-19* and *mammaglobin* were custom synthesised by Applied Biosystems and are listed elsewhere (Benoy *et al*, 2004). Commercially available probes and primer sets for ACTB and TBP were used for normalisation (Applied Biosystems). Fluorogenic PCR analyses were carried out in a reaction volume of 25 *μ*l and contained 12.5 *μ*l of *TaqMan* Gene Expression Master Mix (Applied Biosystems) and 10 *μ*l of cDNA solution. Each sample was analysed in duplicate and mean *C*_t_ values were used for further analysis.

#### Quantification

*C*_t_ values for *CK-19* were normalised for ACTB and TBP expression levels and expressed in relation to a positive control sample using the 2^−ΔΔ*C*t^ quantification method (Livak and Schmittgen, 2001). We use the abbreviation RGE (relative gene expression) to indicate these measurements. RGE was then normalised according to the following equation: 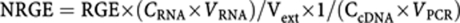 where NRGE is the normalised RGE expressed as relative target concentration per ml of blood; *C*_RNA_ the concentration of total RNA extracted per sample; *V*_RNA_ the elution volume of RNA obtained after extraction; *V*_ext_ the volume of blood extracted; *C*_cDNA_ the concentration of cDNA and *V*_PCR_ the volume of cDNA solution used for PCR amplification.

### Statistical analysis

The Mann–Whitney *U-*test was used to assess differences between non-parametrically distributed variables. Correlations between continuous non-parametric variables were assessed by calculating Spearman's rank correlation coefficient or by the *κ*-test in the case of categorical variables (Landis and Koch, 1977). Differences in rates of positive samples between the three CTC detection methods were investigated using the McNemar test. The Pearson's *χ*^2^-test or, in the case of low frequencies per cell, the Fisher's exact method was used to assess the relationship between rates of positive samples and patient characteristics. A two-sided *P*⩽0.05 was considered to be statistically significant. All statistical calculations were performed using SPSS, version 11.0 (SPSS, Chicago, IL, USA).

## Results

### Detection of CTCs with the CellSearch CTC test

**Figure 1:**
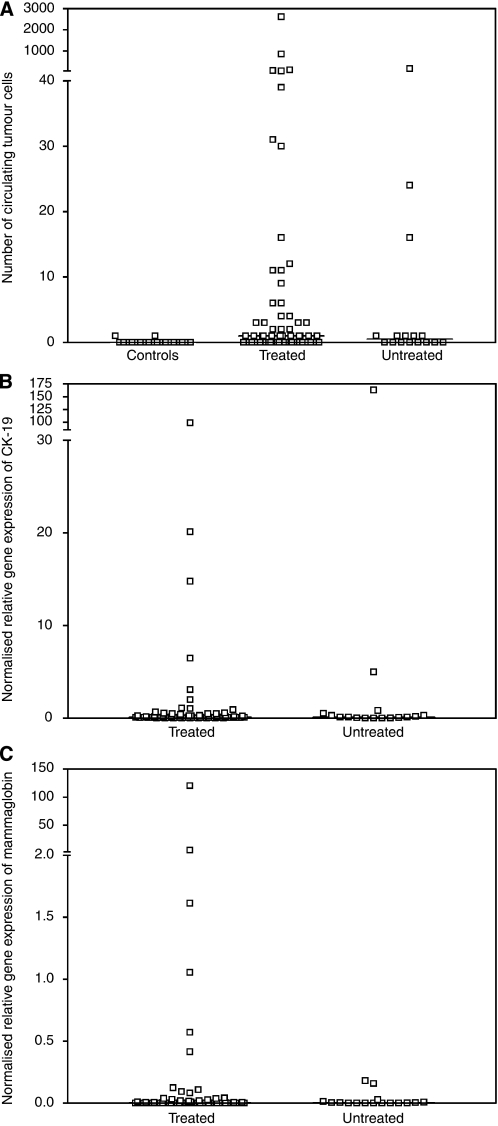
(**A**) Number of CTCs detected in 7.5 ml of blood taken from healthy controls (*N*=20) and metastatic breast cancer (MBC) patients (*N*=76) when samples were analysed with the CellSearch CTC test. (**B**) Normalised relative gene expression levels of CK-19 detected in blood from MBC patients (*N*=76) when samples were analysed using a real-time RT-PCR assay. (**C**) Normalised relative gene expression levels of mammaglobin detected in blood from MBC patients (*N*=76) when samples were analysed using a real-time RT-PCR assay. Treated: patients with MBC during treatment. Untreated: patients with MBC receiving no treatment.

The CellSearch CTC System was used to enumerate CTCs in blood of 76 MBC patients and 20 healthy volunteers. In this study, 59% of MBC patients and 10% of healthy controls had at least one detectable CTC in 7.5 ml of blood (*P*<0.001, Pearson's *χ*^2^-test). For one patient, the sample quality did not permit an accurate CTC enumeration. Numbers of CTC were significantly higher in blood samples of patients with MBC than in healthy controls: the median number of CTCs detected in 7.5 ml of blood was 1 (range, 0–2617) in MBC patients (*N*=75) and 0 (range, 0–1) in controls (*N*=20) (*P*<0.001, Mann–Whitney *U*-test) (Figure 1A). Numbers of CTC did not differ between treated and untreated patients (*P*=0.302, Mann–Whitney *U*-test): the median number of CTCs in the treated patient group (*N*=59) was 1 (0–2617) and the median number of CTCs in the untreated patient group (*N*=16) was 0.5 (0–153). The number of patient samples reaching the cutoff level of 2 or more CTCs in 7.5 ml of blood was 36%. Using this approach, we obtained positive CTC test results by the CellSearch System in 41% of treated patients and in 19% of untreated patients (*P*=0.427, Pearson's *χ*^2^-test).

Next, we assessed the correlation between CTCs in blood and tumour progression (Table 1). The median number of CTCs was 1 (range, 0–2617) in patients with progressive disease (*N*=46) and 0 (range, 0–39) in non-progressive patients (*N*=26) (*P*=0.004, Mann–Whitney *U-*test). Furthermore, a positive correlation between the number of CTCs and serum CA15.3 levels (*r*=0.669, *P*<0.001) or patients’ age was observed (*r*=0.376, *P*=0.001). No associations were found between the presence of two or more CTCs and ER, PR, HER2 or P53 expression by the primary tumour.

### Detection of CTCs with the AdnaTest Breast Cancer Select/Detect

The AdnaTest Breast Cancer Detect is considered positive if a PCR fragment of at least one tumour-associated transcript (*Muc-1*, *GA733-2* or *HER2*) is clearly detected (peak concentration of >0.30 ng *μ*l^−1^ with the BioAnalyzer 2100), it is considered inconclusive if peaks have an intermediate concentration (0.15–0.30 ng *μ*l^−1^) and as negative if peak concentrations are <0.15 ng *μ*l^−1^. Using these criteria, 18 volunteers in the healthy control population (*N*=20) were diagnosed as negative by the AdnaTest, 1 healthy control had a single positive blood sample and 1 healthy control had inconclusive test results. Of 76 MBC patients, 16 patients (22%) had two positive blood samples, 7 patients (9%) had only one positive blood sample and 51 patients (68%) had no positive blood samples. For two patients, an inconclusive test result was observed and these were left out for further analysis. The frequency of a positive test result (two positive blood samples) was significantly higher in MBC patients than in healthy controls, 22 *vs* 0% (*P*=0.03, Pearson's *χ*^2^-test). No significant differences were observed between treated and untreated patient groups.

A positive outcome with the AdnaTest was associated with high CA15.3 levels (relative to the median level in the patient population) (*P*=0.001, Pearson's *χ*^2^-test) but not with tumour progression (*P*=0.22, Pearson's *χ*^2^-test). In addition, no association was found between tumour progression and the number of positive blood samples (*P*=0.35, Pearson's *χ*^2^-test). The outcome of the AdnaTest correlated with the ER status of the primary tumour (*P*=0.02, Pearson's *χ*^2^-test), but not with PR, HER2 or P53 status. Data are summarised in Table 1.

### Detection of CTCs with a quantitative multimarker RT–PCR assay

Sensitivity and specificity of the quantitative real-time RT-PCR assay for *CK-19* and *mammaglobin* have previously been described (Benoy *et al*, 2004). The median NRGE levels in MBC patients (*N*=76) were 0.105 (range, 0–163.15) for *CK-19* and 0.001 (range, 0–120.44) for *mammaglobin* (Figure 1B and C). No significant differences in median NRGE levels for *CK-19* or *mammaglobin* were observed between treated and untreated patient groups (*P*=0.87 and 0.91, Mann–Whitney *U*-test). The median NRGE levels of both mRNA markers were significantly correlated (*r*=0.321, *P*=0.005). NRGE levels of *CK-19* or *mammaglobin* correlated with CA15.3 levels (*r*=0.481, *P*<0.001 and *r*=0.386, *P*=0.001), but not with patients’ age.

We also analysed the PCR results for *CK-19* as categorical variables, by establishing a cutoff value for positivity that corresponded to 100% specificity (maximal NRGE values measured in the control population). On this basis, 20 of 76 (26%) patients were positive for *CK-19*. As no PCR signals for *mammaglobin* were detected in any of the control samples, a patient sample was considered positive when a PCR signal for *mammaglobin* was detected. *Mammaglobin* expression was measurable in 41 of 76 (54%) MBC patients. Of the 76 blood samples from MBC patients, 14 (18%) were positive for both *CK-19* and *mammaglobin*, 33 (43%) were positive for only one mRNA marker and 29 (38%) were negative for both *CK-19* and *mammaglobin*. A positive result for *CK-19* and/or *mammaglobin* mRNA expression was found in 69% of untreated patients (*N*=16) and in 60% of treated patients (*N*=60) (*P*=0.52, Pearson's *χ*^2^-test). There was no significant concordance between samples either positive or negative for *CK-19* and *mammaglobin* (*κ*=0.16, *P*=0.09, *κ*-test).

Next, we analysed the relationship between CTC detected by RT–PCR for *CK-19* or *mammaglobin* and tumour progression (Table 1). The detection rate of *CK-19+* or *mammaglobin*+ blood samples was not associated with tumour progression (*P*=0.19 and 0.84, Pearson's *χ*^2^-test). No associations were found between *CK-19* or *mammaglobin* expression and any of the clinicopathological variables (ER, PR, HER2 and P53).

### Comparison between the three CTC detection methods

**Figure 2:**
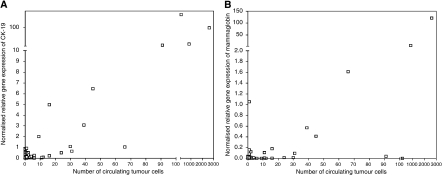
Correlation between the number of circulating tumour cells by the CellSearch System and the normalised relative gene expression levels of CK-19 (**A**) or mammaglobin (**B**) by RT–PCR in MBC patients.

Quantitatively, higher CTC numbers by the CellSearch System and higher NRGE levels for *CK-19* were observed in blood samples defined as positive by the AdnaTest than in negative samples (*P*<0.001 and 0.002, Mann–Whitney *U-*test). For *mammaglobin* expression, no differences were observed (*P*=0.09, Mann–Whitney *U-*test). Furthermore, we observed a good correlation between the number of CTCs detected with the CellSearch CTC test and the NRGE levels of *CK-19* (*r*=0.453, *P*<0.001) or *mammaglobin* (*r*=0.477, *P*<0.001) by RT–PCR (Figure 2).

According to the McNemar test, a significant difference in positivity was observed by the CellSearch CTC test and the AdnaTest (36 *vs* 22%, *P*=0.013). Patients with MBC were more likely to be positive for *CK-19* and/or *mammaglobin* while using real-time RT–PCR than using the CellSearch CTC test (63 *vs* 36%, *P*=0.001, McNemar test) or the AdnaTest (61% *vs* 22%, *P*<0.001, McNemar test).

Concordant samples were defined as those in which the sample from a patient was reported as either positive or negative by both the detection techniques being compared. As a result, the concordance between samples analysed by the CellSearch CTC test and the AdnaTest was moderate (*κ*=0.543, *P*<0.001, *κ*-test). Agreement between both detection techniques was observed in 81% of blood samples. When the CellSearch CTC test was compared with RT–PCR assays for *CK-19* and *mammaglobin*, we observed agreement percentages of 72 and 60%, respectively (*κ*=0.356, *P*=0.002 and *κ*=0.220, *P*=0.04, *κ*-test). Agreement between the AdnaTest and RT–PCR assays for CK-19 and mammaglobin was observed in 78 and 53% of blood samples, respectively (*κ*=0.415, *P*<0.001 and *κ*=0.082, *P*=0.375, *κ*-test). Data are summarised in Table 2. For eight MBC patients (10%), a positive test result was obtained using the three methods.

## Discussion

Despite important advances in the early diagnosis and treatment, metastatic disease occurs in about 50% of cases with apparently localised breast cancer, and even 30% of patients with node-negative disease will develop distant metastases (Braun *et al*, 2001). Staging of carcinoma patients in clinical practice is based on tumour characteristics such as tumour size, tumour grade, lymphovascular involvement, the presence of metastases in regional lymph nodes at the time of primary surgery, steroid receptor status and human epidermal growth factor receptor 2 amplification (Singletary *et al*, 2002). The assessment of CTCs in peripheral blood samples is not considered to be a routine procedure in the clinical management of breast cancer for several reasons (Riethdorf *et al*, 2007). Most notably, the variable technical approaches used, the high inter-laboratory differences in the number of millilitres of blood analysed, the quality of sensitivity and specificity tests, the number of patients *vs* controls and data interpretation make it very difficult to draw firm conclusions about the impact of CTC detection in cancer prognosis and follow-up (Paterlini-Brechot and Benali, 2007). The primary objective of this study was to compare three different techniques for the detection of CTC in blood from patients with MBC: (1) the CellSearch System, which represents an automated, standardised and regulatory-approved system for the immunocytochemical detection and quantification of CTCs in blood; (2) the AdnaTest Breast Cancer Select/Detect, which involves the detection of tumour-associated transcripts by RT–PCR after an immunomagnetic enrichment of tumour cells and (3) an in-house developed multimarker real-time RT-PCR assay, which involves the quantification of tumour-associated transcripts by real-time RT-PCR after enrichment of peripheral blood mononuclear cells by filtration. Technical details of the three detection techniques are summarised in Table 3.

We observed significant differences in the detection frequencies of CTCs among the three assays. Using the CellSearch CTC test, we found that the number of CTCs in our survey of MBC patients ranged from 0 to 2617. In 59% of blood samples, at least one CTC was detected and in 36% of blood samples, two or more CTCs were detected. In contrast, none of the blood samples in the control population contained two or more CTCs, which corresponds to a specificity of 100%. Using the same cutoff value, Allard *et al* (2004) obtained a positivity rate of 37% in a pooled analysis of 1316 blood samples obtained from 422 patients at different occasions. However, when analysing treated and untreated patients separately, only 19% of the untreated patients (*N*=16) included in our study had two or more CTC per 7.5 ml blood, which is considerably lower than the numbers found by Cristofanilli *et al* (2004) in 177 patients with MBC before the start of a new line of treatment (61%) (Table 4). The reason for this discrepancy remains unclear but could be because of the small sample size of untreated patients in our study. When using the AdnaTest, none of the blood samples of healthy subjects were found to be positive. A relatively low rate of two positive blood samples (22%) was observed in the patient population. For comparison, Zieglschmid *et al* (2007a) reported the presence of CTCs in 69% of blood samples from MBC patients using the same technique. However, in a recent study by Aktas *et al* (2009) investigating the expression of EMT and stem cell markers in CTCs detected with the AdnaTest, CTCs were found in 69 of 226 (31%) blood samples taken from patients with MBC (Aktas *et al*, 2009; Table 4). Using our previously developed quantitative real-time RT-PCR assay, we measured normalised RGE levels in cancer patients ranging from 0 to 163 for *CK-19* and from 0 to 120 for *mammaglobin*. Low levels of *CK-19* mRNA were also found in the blood of healthy donors. The detection of *CK-19* in healthy donors by RT–PCR has been attributed to an illegitimate transcription of the *CK-19* gene in peripheral blood mononuclear cells (Novaes *et al*, 1997) and/or to an increased secretion of cytokines that can induce transcription of tissue-specific genes in peripheral blood leukocytes (Chelly *et al*, 1989; Jung *et al*, 1998). To solve this issue, we chose a cutoff for positivity, which corresponded to a specificity of 100%. On this basis, 26% of patient samples were positive for *CK-19* mRNA. As *mammaglobin* expression was never detected in blood samples from healthy controls, patient samples were called positive when a PCR signal for *mammaglobin* mRNA was detected. On this basis, 54% of patient samples were positive for *mammaglobin* expression. Notably, 61% of patients were positive for at least one of the two transcript markers, which shows the benefit of using more than one target for RT–PCR amplification. The sensitivity of the multimarker real-time RT-PCR assay significantly exceeded that of the CellSearch CTC test and that of the AdnaTest.

Aside from the relative sensitivities and specificities of the different techniques, we were also interested in the correlations between techniques and their concordance. We observed significant correlations between cell numbers detected by the CellSearch System and normalised RGE levels of *CK-19* and *mammaglobin* mRNA by real-time RT-PCR. When a cutoff for positivity was established, the concordance between the CellSearch System and the real-time RT-PCR assay was 72 and 60%, depending on the transcript marker. Overall, the concordances between techniques ranged from 53% (for AdnaTest and *mammaglobin* RT–PCR) to 81% (for AdnaTest and CellSearch System). Thus, samples being called positive or negative differed according to the CTC detection technology. The low concordance between our real-time RT-PCR assay and the AdnaTest may be explained by several reasons: (1) the AdnaTest is based on an immunomagnetic enrichment of tumour cells by epithelial and tumour-associated antigens, whereas in our assay, filter technology is used to remove red blood cells; (2) different RNA isolation and RT protocols are used in both assays; (3) the presence of tumour cells is indicated by different transcript markers and (4) in the AdnaTest, PCR products are visualised using microfluidic gel electrophoresis after which peak concentrations are measured, whereas the real-time PCR assay allows for a more sensitive quantification of mRNA expression levels by which also the exact amount of background transcription can be assessed.

Although, in general, approaches based on RT–PCR have a high sensitivity for the detection of CTC, an important limitation of these methods is that these cannot quantify the number of CTCs and no morphological evaluation of cells can be obtained. Furthermore, it remains unclear whether the minute tumour dissemination detected by PCR is capable of causing clinically relevant distant metastasis. In contrast to RT–PCR assays, the CellSearch CTC test allows for the counting of target cells. Advantages of the CellSearch System are its capability of standardising pre-analytical preparation of CTCs, the use of CTC preservative tubes that allow stabilisation of CTCs for up to 96 h and the inclusion of a positive control for assuring proper performance on a daily or run-to-run basis. All these features are beneficial in a multi-centre setting in clinical trials. One of the limitations of the CellSearch System, however, is the anti-EpCAM antibody-based enrichment strategy. Several authors reported the heterogeneous expression of EpCAM in mammary carcinomas (Thurm *et al*, 2003) and downregulation of EpCAM has been reported for disseminated tumour cells in bone marrow and CTCs in peripheral blood (Thurm *et al*, 2003; Rao *et al*, 2005). Recently, Deng *et al* (2008) showed that CTC enrichment with anti-cytokeratin antibodies, in combination with anti-EpCAM antibodies, significantly enhances assay sensitivity.

Summarising the results of this study, our multimarker quantitative RT-PCR assay showed superior sensitivity for the detection of CTCs in MBC compared with the CellSearch System and the AdnaTest. However, further studies are needed to clarify the prognostic value of this highly sensitive and standardised qRT-PCR approach for CTC detection in peripheral blood of patients with breast cancer.

## Figures and Tables

**Table 1:** Relations of positive rates by the three CTC detection techniques with clinicopathological variables and other molecular markers in blood

	**All patients**		**CellSearch CTC test**			**AdnaTest**			**RT–PCR for CK-19**			**RT–PCR for mammaglobin**			**RT–PCR CK-19 and/or mammaglobin**		
**Characteristic**	***N***	**%**	***N***	**%**	***P***	***N***	**%**	***P***	***N***	**%**	***P***	***N***	**%**	***P***	***N***	**%**	***P***
ER																	
Positive	51	71	21	81	0.20	15	94	**0.02**	15	75	0.63	29	74	0.47	33	73	0.55
Negative	21	29	5	19		1	6		5	25		10	26		12	27	
																	
*PR*																	
Positive	37	53	13	52	0.84	9	60	0.53	9	47	0.57	21	57	0.49	23	53	0.89
Negative	33	47	12	48		6	40		10	53		16	43		20	47	
																	
*HER2*																	
Positive	25	33	7	26	0.36	3	19	0.17	4	20	0.14	13	32	0.74	14	30	0.40
Negative	50	67	20	74		13	81		16	80		28	68		33	70	
																	
*P53*																	
Positive	18	37	6	35	0.89	3	25	0.39	8	53	0.11	10	36	0.86	13	39	0.58
Negative	31	63	11	65		9	75		7	47		18	64		20	61	
																	
*Disease status*																	
Progressive	46	63	22	81	**0.02**	12	75	0.22	15	75	0.19	25	64	0.84	30	67	0.41
Non-progressive	27	37	5	19		4	25		5	25		14	36		15	33	
																	
*CA15.3 levels* a																	
High (⩾109 U/ml)	34	51	22	85	**<0.001**	14	87	**0.001**	15	823	**0.001**	20	54	0.55	24	56	0.27
Low (<109 U/ml)	33	49	4	15		2	13		3	17		17	46		19	44	

Abbreviations: CTC=circulating tumour cell; ER=oestrogen receptor; PR=progesterone receptor; RT–PCR=reverse transcription–PCR.

Statistically relevant relations are presented in bold.

a Serum CA15.3 levels were dichotomised according to the median level in the breast cancer population.

**Table 2:** Concordances between the three CTC detection techniques

	**K coefficient**	***P*-value**	**Degree of agreement**	**Agreement (%)**
*Comparison with the CellSearch CTC test*				
AdnaTest	0.543	<0.001	Moderate	81
RT–PCR for CK-19	0.356	0.002	Fair	72
RT–PCR for mammaglobin	0.220	0.04	Fair	60
RT–PCR for CK-19 and/or mammaglobin	0.203	0.04	Fair	57
				
*Comparison with the AdnaTest*				
RT–PCR for CK-19	0.415	<0.001	Moderate	78
RT–PCR for mammaglobin	0.082	0.37	Slight	53
RT–PCR for CK-19 and/or mammaglobin	0.109	0.189	Slight	50

Abbreviations: CTC=circulating tumour cell; ER=oestrogen receptor; PR=progesterone receptor; RT–PCR=reverse transcription–PCR.

**Table 3:** Comparison of the three detection techniques

**Assay**	**Blood volume per test**	**Principle of CTC enrichment**	**Method of detection**
CellSearch	7.5 ml	Immunomagnetic – EpCAM	Visual confirmation of fluorescently labeled cells (CK+/DAPI+/CD45−)
AdnaTest	2 × 5 ml	Immunomagnetic – 2 anti-Muc-1 Ab and 1 anti-EpCAM Ab	Multiplex RT–PCR for *HER2*, *Muc-1* and *EpCAM*
CK-19/MAM RT–PCR	9 ml	Size filtration of PB mononuclear cells	Multiplex real-time quantitative RT–PCR for *CK-19* and *mammaglobin*

Abbreviations: CTC=circulating tumour cell; RT–PCR=reverse transcription–PCR.

**Table 4:** Comparison of positivity rates for CTC detection in MBC

**Reference**	**Method**	**Volume (ml)**	**No. of patients**	**No. of samples**	**Positivity rate (% of samples)**
Allard *et al* (2004)	CellSearch	7.5	422	1316	37^a^
Cristofanilli *et al* (2004)	CellSearch	7.5	177	NA	61^a^
Zieglschmid *et al* (2007a)	AdnaGen	2 × 5	48	NA	69
Aktas *et al* (2009)	AdnaGen	2 × 5	39	226	31

Abbreviation: NA=not applicable.

a Positivity rate defined as 2 or more CTC per 7.5 ml peripheral blood.
